# Cryo-EM Structure of Siphophage Avs-1 Reveals a Conserved Virion Architecture with a Specialized Tripod-like Tail Tip for Host Recognition

**DOI:** 10.3390/v18091011

**Published:** 2026-09-14

**Authors:** Jiayi Liu, Chen Li, Xiaorong Yang, Fan Yang, Hongrong Liu

**Affiliations:** 1 Key Laboratory for Matter Microstructure and Function of Hunan Province, School of Physics and Electronics, Hunan Normal University, Changsha 410081, China; 2 Institute of Interdisciplinary Studies, Hunan Normal University, Changsha 410081, China; 3 Hunan Provincial Key Laboratory of Microbial Molecular Biology, College of Life Science, Hunan Normal University, Changsha 410081, China

**Keywords:** *Aeromonas veronii*, siphophage, cryo-electron microscopy, tail tip structure, aquaculture

## Abstract

Bacteriophages are increasingly considered viable alternatives to antibiotics for controlling aquaculture pathogens, yet the structural basis of siphophage infection in aquatic bacteria remains largely unexplored. Here, we present the near-atomic-resolution cryo-electron microscopy structure of Avs-1, a siphophage infecting the freshwater aquaculture pathogen *Aeromonas veronii*. The capsid reconstruction at 2.98 Å and spike structure at 3.58 Å reveal a virion architecture broadly conserved among lambda-like siphophages, including an HK97-fold capsid, a canonical portal–neck complex, and a tail tube composed of approximately 26 stacked hexameric rings. However, the distal tail tip exhibits pronounced specialization, assembling into a C3-symmetric tripod-like core formed by the distal tail protein, hub protein, insertion protein, and the C-terminus of the tape measure protein. The trimeric spike protein gp43 extends from this platform and adopts a β-helical scaffold with a structurally divergent distal domain (D4), highlighting the structural diversity of phage tail-tip architectures. Together, these results reveal how a largely conserved virion framework is combined with a diversified distal tail apparatus, expanding the structural repertoire of characterized siphophages and providing a framework for investigating the molecular basis of phage–host interactions.

## 1. Introduction

Bacteriophages targeting aquaculture pathogens, such as *Aeromonas veronii*, have garnered considerable attention as promising alternatives to conventional antibiotics, particularly given the escalating prevalence of antimicrobial resistance in aquaculture settings [1,2,3]. The infection cycle is initiated by the phage tail apparatus, a multifunctional machine that orchestrates host recognition, cell envelope penetration, and viral genome delivery [4,5,6,7]. In siphophages, the distal tail tip constitutes the primary adsorption machinery, comprising receptor-binding proteins and tail spikes that determine host specificity [6,7,8,9,10,11]. Nevertheless, the structural basis by which siphophages recognize and engage their aquatic bacterial hosts remains largely elusive.

Extensive structural characterization of siphophages infecting terrestrial pathogens has revealed a conserved architectural framework for the tail tip, punctuated by remarkable diversity in peripheral receptor-binding components that underpin host specificity [7,9,10,11,12,13,14,15,16]. A notable example is the β-helical tailspike, which commonly serves as a scaffold for receptor interactions in phages such as Sf6 [11], CBA120 [10], P22 [7], and GVE2 [9], where it is coupled to distal modules that confer functional specialization. However, high-resolution structures of phages infecting aquaculture pathogens remain scarce. Importantly, the cell-surface properties of aquatic bacteria differ markedly from those of terrestrial pathogens, particularly in the composition and structural plasticity of their lipopolysaccharides and extracellular polysaccharide layers, which are shaped by the more dynamic and variable environmental conditions prevalent in aquatic habitats [17]. These fundamental differences suggest that structural insights derived from terrestrial phage models may not be directly translatable to their aquatic counterparts. *Aeromonas veronii*, a major freshwater aquaculture pathogen responsible for substantial economic losses worldwide [18,19,20], thus represents a critical target for such structural investigation.

In our recent study, we isolated and characterized phage Avs-1, a virulent siphophage targeting the freshwater aquaculture pathogen *Aeromonas veronii*, and obtained initial cryo-EM reconstructions of its major structural regions [21], whereas the molecular basis underlying the organization and diversification of its structural modules remained unclear. Here, we extend these findings by presenting high-resolution cryo-EM structures and constructing atomic models of the Avs-1 virion and performing detailed structural analyses of its major components. Our structure reveals a conserved siphophage tail architecture assembled with a specialized distal adsorption system, wherein the distal tail protein, hub protein, insertion protein, the C-terminus of the tape measure protein, and the trimeric spike coalesce in a C3-symmetric tripod-like organization. The spike region, resolved to 3.58 Å, enabled confident atomic modeling of the β-helical scaffold and the putative receptor-binding region. Collectively, these findings provide a high-resolution structural framework for understanding phage–host interactions and the structural diversity of siphophage distal tail modules.

## 2. Materials and Methods

### 2.1. Culture and Purification of Avs-1

The Avs-1 strain used in this study was derived from the isolate described previously [21]. For cryo-EM structural analysis, Avs-1 particles were independently propagated and purified using a dedicated purification procedure described below. *Aeromonas veronii* AV1212 was cultured overnight in Luria–Bertani (LB) broth (5 g/L yeast extract, 10 g/L tryptone, and 10 g/L NaCl) at 30 °C. Avs-1 phages were next incubated with the bacterial culture for 8 h at 30 °C. Subsequently, the cell debris was removed by centrifugation at 7200× *g* for 30 min at 8 °C. Phage particles in the supernatant were precipitated overnight at 4 °C using 1 M NaCl and 10% (*w*/*v*) PEG 8000. The precipitated phages were recovered by centrifugation at 11,000× *g* for 45 min and resuspended in phosphate-buffered saline (PBS). Phages were then purified by ultracentrifugation through a CsCl step gradient (1.2 g/mL, 1.3 g/mL, 1.4 g/mL, 1.5 g/mL, and 1.6 g/mL) at 128,600× *g* for 2 h at 8 °C. The visible phage-containing band was collected and dialyzed against PBS at 4 °C overnight to remove CsCl. Finally, the purified Avs-1 phages were stored on ice prior to cryo-electron microscopy sample preparation.

### 2.2. Cryo-EM Sample Preparation and Data Collection

The purified phage Avs-1 aliquot (3.5 µL) was pipetted onto graphene oxide (GO) grids that had been glow-discharged for 30 s. The grids were then loaded into a Thermo Fisher Scientific (TFS) Vitrobot (Waltham, MA, USA) set to 100% humidity and blotted for 3.5 s at 8 °C. After blotting, the grids were vitrified by plunging into liquid ethane and subsequently stored in liquid nitrogen. Data collection was performed using a TOTEM ARM 300s transmission electron microscope equipped with a K3 direct electron detector (Gatan, Pleasanton, CA, USA) at a magnification of 40,000×, yielding a calibrated physical pixel size of 1.248 Å. Each movie was exposed to a total dose of approximately 32 e^−^/Å^2^, and a total of 3068 micrographs were collected, with each movie stack comprising 32 image frames.

### 2.3. Image Processing and 3D Reconstruction

All image processing and three-dimensional reconstruction procedures were performed using the cryoSPARC v4.6.0 software platform [22]. The workflow began with contrast transfer function (CTF) correction on 3068 motion-corrected micrographs. From 2363 micrographs, Avs-1 head particles were automatically picked using the Blob Picker. Subsequent 2D classification was performed to select homogeneous particles, yielding 43,948 particles extracted with an 800 × 800 pixel box size. These particles were subjected to ab initio reconstruction followed by homogeneous refinement, both with imposed icosahedral (I) symmetry. This procedure yielded a final cryo-EM density map of the DNA-filled capsid at a global resolution of 2.98 Å. These particles were then symmetry expanded according to the I symmetry for further localized processing. The center of the reconstructed map was subsequently shifted to a vertex of the icosahedral capsid using the Volume Alignment Tools. Duplicate particles were removed using the Remove Duplicates job. Following the initial particle extraction, 3D classification was performed to select a subset of 33,454 particles containing the portal-containing vertex, using a box size of 320 × 320 pixels. These particles were refined with C6 symmetry using homogeneous refinement, which resulted in a reconstruction of the neck region at a resolution of 3.12 Å. To reconstruct the tail tube structure, the volume was recentered along the longitudinal axis toward the tail region using the Volume Alignment Tools. Based on this adjusted volume center, 33,745 particles were re-extracted using a box size of 230 × 230 pixels. An initial reconstruction of the tail tube was generated using the Reconstruct Only job and subsequently refined under C3 symmetry, producing a density map of the tail tube at a resolution of 3.30 Å.

To reconstruct the Avs-1 tip, particles were initially manually picked to generate templates for subsequent automated particle picking via the Template Picker. This process yielded 22,840 particles, which were extracted using a box size of 350 × 350 pixels. Following 2D classification to remove non-target particles and ensure homogeneity, an initial volume was generated through ab initio reconstruction. The model was then subjected to iterative rounds of homogeneous refinement, local refinement, and local CTF refinement, ultimately resulting in a tip structure at a resolution of 3.62 Å.

Particles from the refined tip map were subjected to symmetry expansion based on C6 symmetry. The center of the expanded particles was then shifted to an individual spike protein using the Volume Alignment Tools, followed by particle extraction, resulting in a total of 137,034 particles extracted with a box size of 240 × 240 pixels. An initial volume was generated using the Reconstruct Only job in C1 symmetry, and subsequent local refinement yielded a density map of the spike region at a resolution of 3.58 Å.

### 2.4. Model Building and Refinement

Atomic models of the Avs-1 virion proteins were built based on the cryo-EM density maps using a combination of automated prediction and manual refinement. Initial backbone tracing and model construction were performed with ModelAngelo [23], which generated de novo atomic models for the major capsid protein (MCP) gp8, portal protein gp6, adaptor protein gp11, stopper protein gp12, tail terminator protein gp14, tail tube protein (TTP) gp15, distal tail protein (DTP) gp20, hub protein gp23, insertion protein gp21, spike protein gp43, and the C-terminal region of the tape measure protein (TMP) gp19.

The preliminary models generated by ModelAngelo were subsequently inspected and manually corrected in Coot v0.9.4 [24]. Residue registration, side-chain fitting, and local conformations were adjusted according to the cryo-EM densities, particularly in flexible regions and at protein–protein interfaces. Regions with weak or discontinuous density, including flexible terminal segments and disordered loops, were either adjusted conservatively or excluded from final model building. Domain boundaries and structural assignments were further validated through structural comparison with homologous proteins and DALI analysis, followed by real-space refinement using PHENIX v1.19.2 [25]. Model quality was evaluated using standard stereochemical and cryo-EM validation metrics implemented in PHENIX and Coot. Final refinement statistics and validation parameters are summarized in Table S2.

## 3. Results and Discussion

### 3.1. Overall Structure of Avs-1 Phage

The isolation, biological characterization, genome sequencing, and initial structural reconstructions of Avs-1 have been reported in our recent companion study [21], revealing its biological potential and providing an overview of its virion architecture. Here, we extend this work by building atomic models for all the major structural components and analyzing the organization and interactions among these proteins. The Avs-1 isolate described previously was propagated in *A. veronii* AV1212 and purified for cryo-EM analysis [21] (Figure S1A). From 3068 micrographs, a total of 44,943 particles were picked. Using cryoSPARC [22], we obtained three-dimensional reconstructions of the major structural regions of Avs-1 ranging from 2.98 Å to 3.62 Å (Figure S1B). Local resolution maps spanning the entire virion from head to tail tip were generated using ResMap-1.1.4 [26] (Figure S2). These maps permitted de novo atomic model building of the major structural proteins, including major capsid protein (MCP) gp8, portal protein gp6, adaptor protein gp11, stopper protein gp12, tail terminator protein gp14, tail tube protein gp15, distal tail protein gp20, hub protein gp23, insertion protein gp21, spike protein gp43, and the C-terminal region of the tape measure protein (TMP) gp19. In contrast, the N-terminus of TMP remained unresolved, presumably owing to its inherent flexibility (Figure S3A,B). 

**Figure 1:**
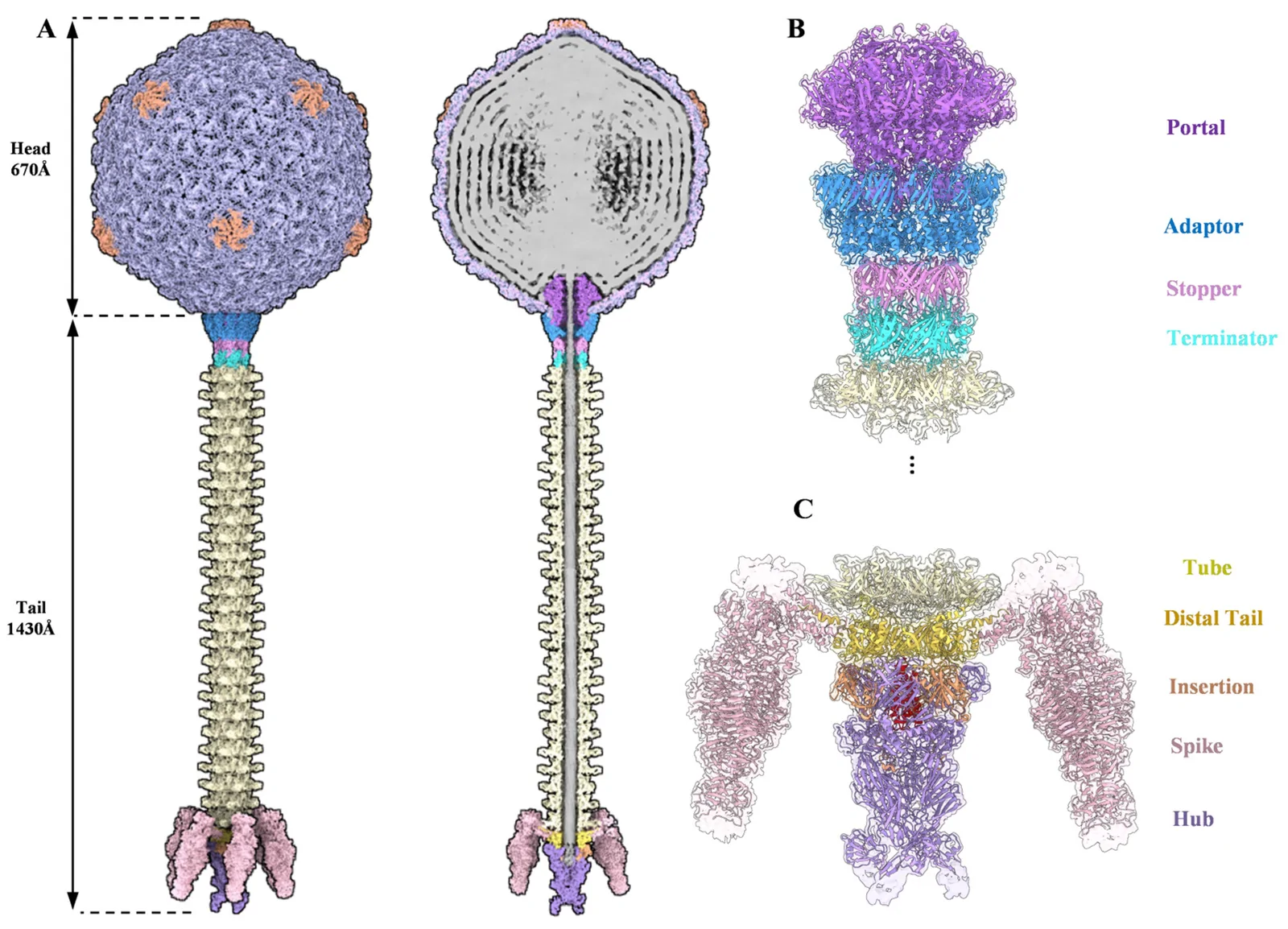
Overall architecture of siphophage Avs-1: (**A**) Side (**left**) and cut-open (**right**) views of the asymmetric structure of the Avs-1 virion. (**B**) Side view of the ribbon models of the connector (portal protein gp6, adaptor protein gp11, and stopper protein gp12) and the tail tube (tail terminator protein gp14 and tail tube protein gp15). (**C**) Side view of the ribbon models of the tail tip components, comprising distal tail protein gp20, insertion protein gp21, hub protein gp23, and spike protein gp43.

The Avs-1 virion measures approximately 2100 Å in total length, with the tail accounting for about 1430 Å (Figure 1A). The capsid adopts the canonical HK97-fold [27], forming a T = 7 icosahedral shell (Figure S4). The portal–neck complex comprises the portal protein gp6, which forms a dodecameric channel at a unique capsid vertex, together with the adaptor protein gp11 and the stopper protein gp12 (Figure 1B). These components mediate the transition between the capsid and the tail. The tail tube is organized by the tail terminator protein gp14 and comprises approximately 26 stacked hexameric rings of tail tube protein gp15, which assemble around the trimeric tape measure protein (TMP) gp19. At the distal end of the tail, the tail tip core is formed by distal tail protein gp20, hub protein gp23, insertion protein gp21, and the C-terminal region of TMP, which together assemble into a C3-symmetric tripod-like structure. From this platform, the trimeric spike protein gp43 extends outward to mediate host recognition (Figure 1C).

These structural analyses establish an overall view of the Avs-1 virion architecture and provide the basis for examining the organization and interactions of individual structural modules. Detailed comparisons with previously characterized siphophages are discussed in the following sections.

### 3.2. Structure of the Portal–Neck Complex

**Figure 2:**
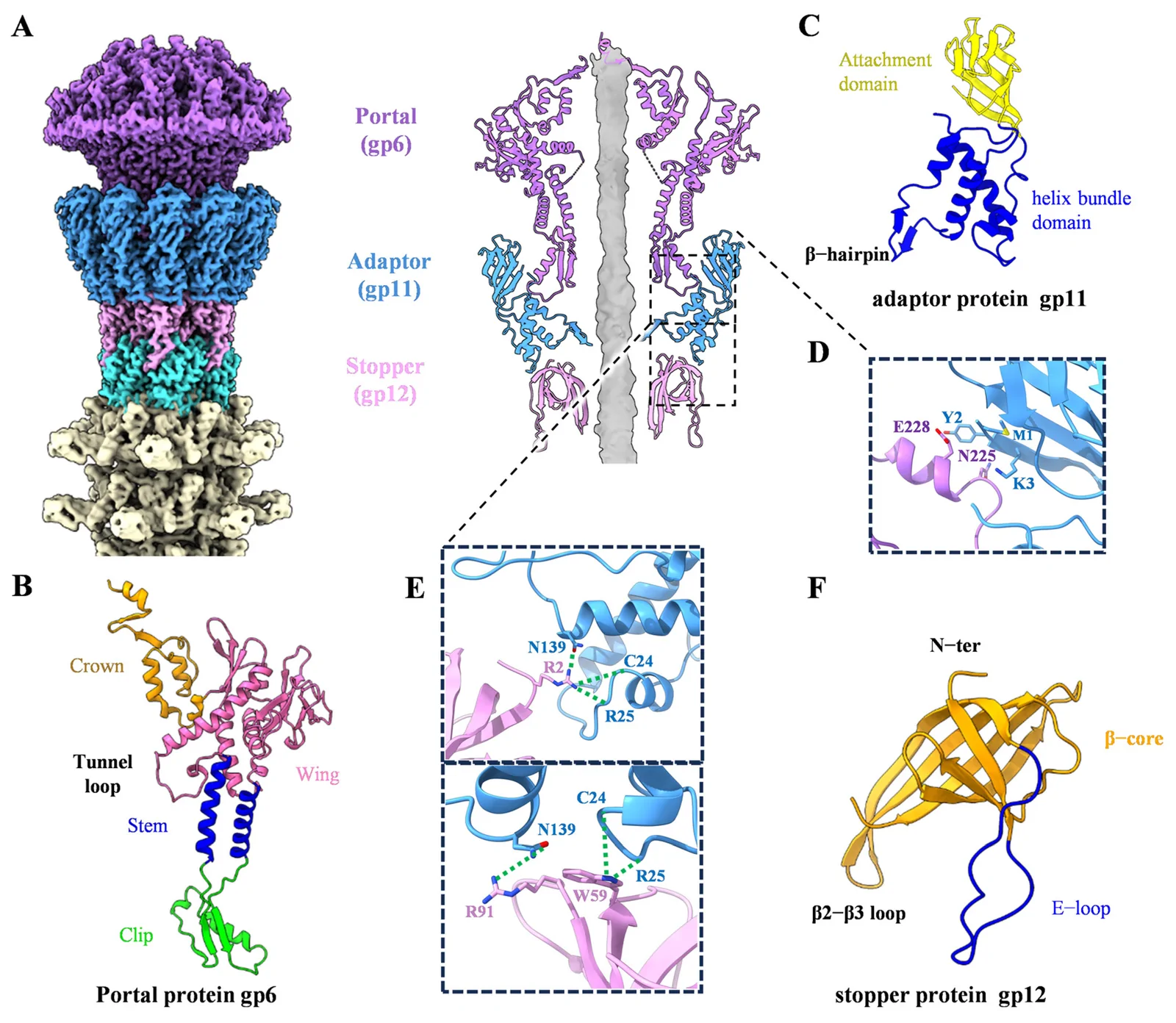
Structure of the connector complex: (**A**) Side view (**left**) of the density maps and slab view (**right**) of the ribbon models of the connector complex. (**B**) Ribbon models of the portal protein gp6, colored according to their domains. (**C**) Ribbon models of the adaptor protein gp11, with distinct domains shown in different colors. (**D**) Interactions between the portal and adaptor subunits, colored consistently with Fig. 2A. (**E**) Interactions between the adaptor and stopper, colored according to their domains. (**F**) Ribbon models of the stopper protein gp12, colored according to their domains.

Positioned at a unique fivefold vertex of the capsid, the portal–neck complex of Avs-1 forms a continuous channel that connects the capsid interior to the tail tube (Figure 2A). The portal, composed of twelve copies of gp6, adopts the canonical Caudoviricetes portal architecture that is conserved among siphophages [28,29,30,31]. In line with the portal proteins of phages lambda [6], DT57C [32], NF5 [14], and R4C [29], gp6 comprises four structural domains. Regions with weak or discontinuous density, most likely attributable to intrinsic flexibility, were excluded from the final model. These include the wing (residues 42–187, 283–337), stem (residues 188–205, 263–282), clip (residues 206–262), and crown domains (residues 338–402) (Figure 2B and Figure S5A). The overall fold is highly conserved despite sequence divergence, with local variation mainly in the peripheral regions of the wing and crown domains. (Figure S5A).

Positioned beneath the portal, the neck contains a dodecameric adaptor ring and a hexameric stopper ring (Figure 2A). Following the domain nomenclature of DT57C head completion protein (HCP) [32] and RcGTA g6 [33], each gp11 monomer can be divided into two domains: an attachment domain (residues 1–8, 56–118) and an α-helical bundle domain (residues 9–55, 119–168) (Figure 2C). The dodecameric adaptor ring comprises an attachment domain and a helical bundle domain, which contact the portal and stopper, respectively (Figure 2D,E). Its organization resembles the head completion protein of DT57C, supporting a conserved role in stabilizing the portal–neck interface (Figure S5A).

**Figure 3:**
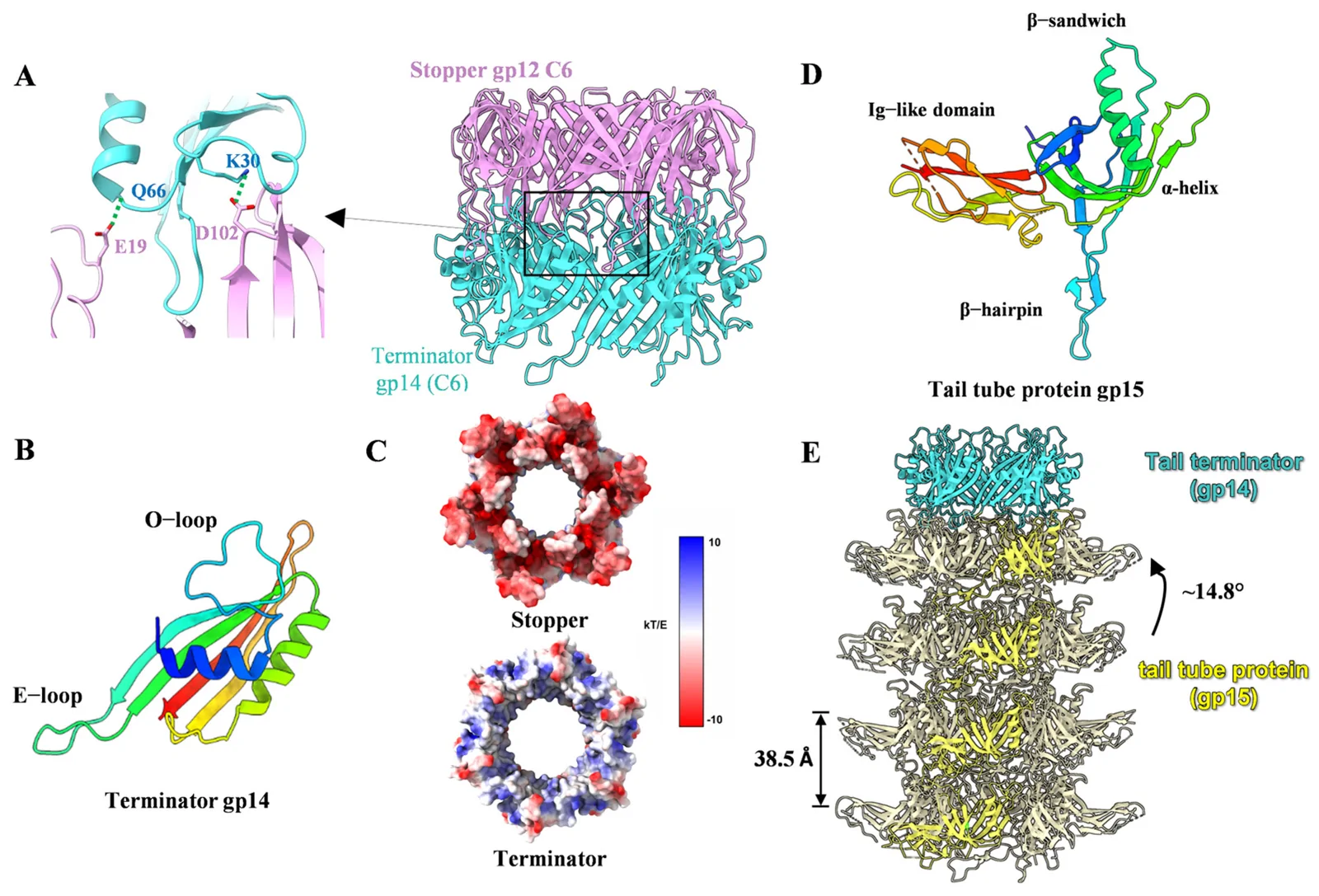
Structure of the tail tube: (**A**) Interactions between the stopper and terminator complex, green dashed lines indicae hydrogen bonds, colored consistently with Fig. 2A. (**B**) Ribbon model of the tail terminator protein gp14, rendered in rainbow coloration from N-terminus (blue) to C-terminus (red). (**C**) Electrostatic potential surface representations of the interface between the stopper and terminator. The upper panel is oriented toward the tip, whereas the lower panel faces the head. The electrostatic potential scale is shown in the color bar. (**D**) Ribbon model of the tail tube protein gp15, colored in the rainbow spectrum. (**E**) Ribbon models of the terminator and four stacked tail tube protein (TTP) rings, with one helical TTP strand highlighted in yellow. The helical rise and twist are indicated.

Below the adaptor ring lies the hexameric stopper gp12, which mediates the symmetry transition from twelvefold to sixfold. Consistent with the organization of NF5 gp9, the Avs-1 stopper comprises a central β-barrel core, a β2-β3 loop, and a β-hairpin domain (Figure 2F). Analogous to NF5 [14], six gp12 subunits oligomerize into a ring that extends the neck lumen and presents a wedge-shaped surface for tail attachment (Figure 3A). 

The overall organization of the Avs-1 portal–neck complex closely resembles the conserved head–tail connector architecture observed in terrestrial siphophages. Thus, siphophages infecting aquaculture pathogens appear to preserve the fundamental structural framework required for head–tail assembly while allowing variation in peripheral regions.

### 3.3. Structure of the Tail Tube

Extending from the neck region, the Avs-1 tail tube is initiated by a hexameric ring of tail terminator protein gp14, which is succeeded by approximately 26 stacked hexameric rings of the tail tube protein gp15 (Figure 1A). Each gp14 monomer comprises an antiparallel β-sheet core flanked by two α-helices, together with an O-loop (residues 16–30) and an E-loop (residues 42–53) (Figure 3B). The lumen of the tail tube is occupied by the tape measure protein (TMP), which forms a continuous channel that extends from the neck toward the tail tip (Figure 1A). At the neck–tail interface, the distal face of the stopper engages in extensive interactions with gp14 (Figure 3A), while complementary interfaces between the adaptor and stopper thereby stabilize the neck–tail junction and maintain continuity of the DNA-translocation pathway (Figure 3C). The architecture of gp14 is conserved with lambda gpU [34], T5 p142 [30], and SPP1 gp17 [35] (Figure S5B). 

Each gp15 monomer comprises an N-terminal TTP-fold domain and a C-terminal immunoglobulin-like (Ig-like) domain (Figure 3D). The TTP-fold domain constitutes the main framework of the tube, adopting a canonical β-sandwich architecture flanked by an α-helix [36,37,38,39,40], akin to that observed in lambda phages [6] (Figure S5C). The TTP-fold forms the main tube framework, whereas the Ig-like domain projects outward from the tube surface. Similar surface-exposed Ig-like domains have been reported in siphophages, including Lambda [34], JBD30 [15], and Chi [16]. The Avs-1 tube adopts a right-handed helical architecture with a rise of ~38.5 Å and a twist of ~14.8° between adjacent rings (Figure 3E), broadly resembling lambda but with a shorter inter-domain linker that places the Ig-like domain in a more orthogonal orientation relative to the tube axis. These differences represent local variations in tube packing while preserving the conserved overall tail-tube architecture. 

The tail tube of Avs-1 conforms to the conserved structural organization established for lambda-like siphophages, although subtle differences in helical parameters, local packing, and inter-domain arrangement are observed. By comparison, structural diversification becomes more pronounced at the distal tail tip, which represents a distinctive feature of Avs-1 and is examined in the following section.

### 3.4. Structure of the Tail Tip Core

**Figure 4:**
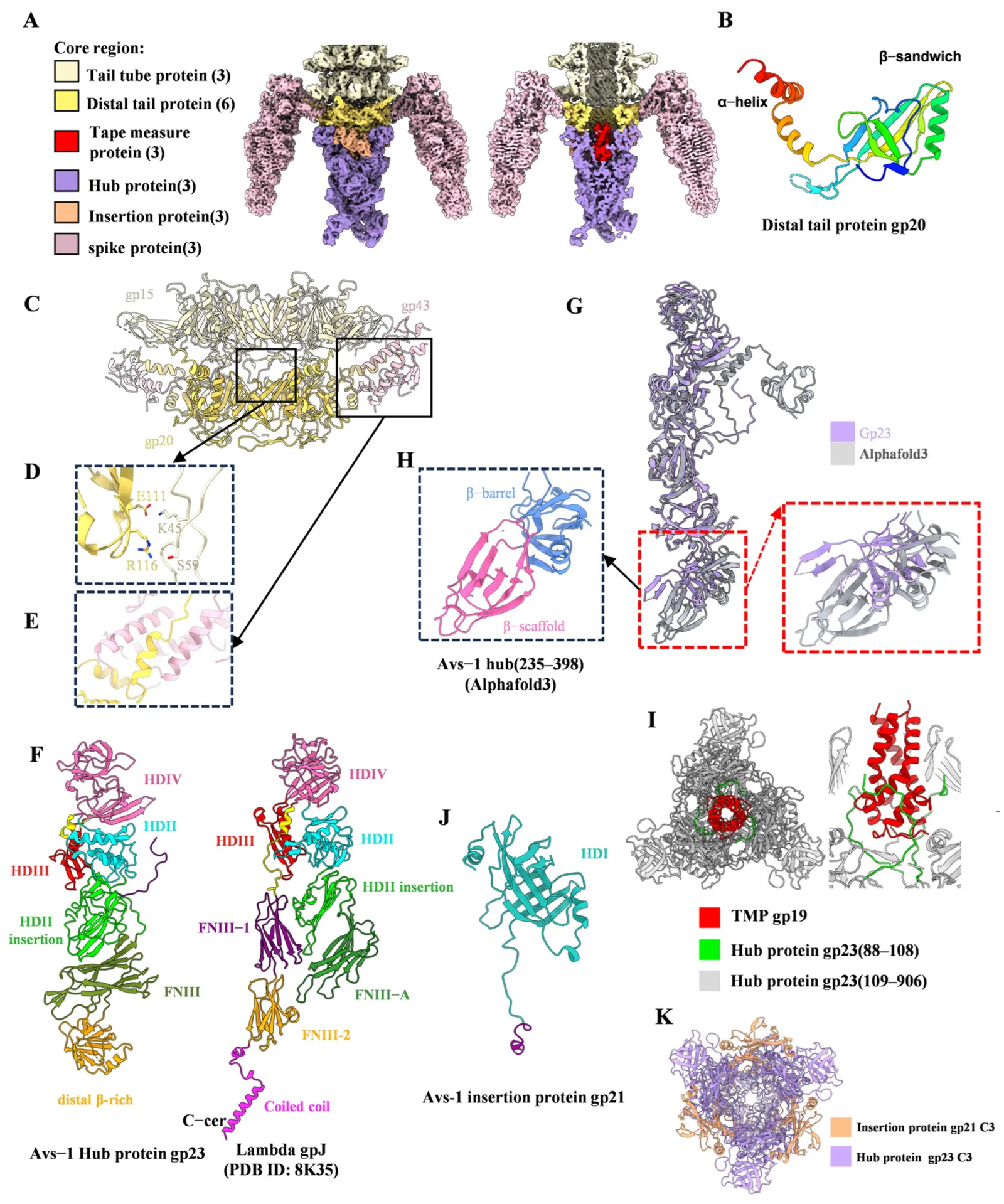
Structure of the core region in the Avs-1 tip: (**A**) Side (**left**) and cut-open (**right**) views of the core region in the tip complex. The core consists of a hexamer of DTP gp20, a trimer of insertion protein gp21, and a trimer of the TMP gp19 C-terminal portion. Color codes are identical to those in Figure 1A. This is a composite map, combining cryo-EM densities of the C6-symmetrized TTP ring, the C6-symmetrized DTP, and the C3-symmetrized tip. (**B**) Ribbon model of the distal tail protein gp20 shown in rainbow colors. (**C**) Side view of a hexameric ring of TTP gp15, a hexameric ring of DTP gp20, and the N-terminal portion of spike protein gp43. (**D**) Magnified view of the interactions between TTP and DTP, colored consistently with Figure 4C. (**E**) Magnified view of the interactions between DTP and spike. (**F**) Structural comparison of the hub protein between Avs-1 and lambda, colored according to their domains. (**G**) Structural comparison of the ribbon models between gp23 and the AlphaFold3 prediction of full-length gp23. (**H**) Ribbon model of the hub protein gp23 (residues 235–398) predicted by AlphaFold3, colored according to its domains. (**I**) Top and side views of trimeric gp19 (red) and gp23 (grey). (**J**) Structure of insertion protein gp21, with distinct domains shown in different colors. (**K**) Top views of trimeric gp21 and gp23.

The core region of the Avs-1 tail tip, located at the distal extremity of the tail tube, comprises four protein components: the distal tail protein (DTP) gp20, the hub protein gp23, the insertion protein gp21, and the C-terminal region of the tape measure protein (TMP) gp19. These components collectively assemble into a C3-symmetric distal tail apparatus (Figure 4A and Figure S6A). DTP gp20 is positioned at the proximal end of the tail tip, immediately beneath the distal ring of the tail tube, where it mediates the transition between the tube and the tip (Figure 4B,C). The β-sandwich domain of gp20 exhibits a high degree of structural conservation with the DTP of Lambda and interacts with the distal ring of the tail tube protein gp15 to secure the tail tip to the distal end of the tail tube (Figure 4D and Figure S6B). An extended α-helical domain projects distally and engages in interactions with the N-terminal helices of the trimeric spike protein gp43, thereby forming a helical bundle that stabilizes the spike within the distal tip assembly (Figure 4E).

The hub protein gp23 constitutes the central structural component of the Avs-1 tail tip. Structural comparison indicates that gp23 shares strong similarity with hub proteins of lambda, even though the sequence identity between the two proteins is merely 14%. According to the domain nomenclature of lambda gpJ [34], gp23 can be divided into six structural domains, namely HDII, HDII insertion, HDIII, HDIV, FNIII, and a distal β-rich domain (Figure 4F). Unlike the needle-like tail tip morphology observed in lambda, the organization of the distal region of Avs-1 gp23 contributes to the formation of a tripod-like tail tip architecture (Figure S6C). Among these, the HDIV, HDIII, HDII, and HDII-insertion domains are highly conserved and display marked similarity to their counterparts in lambda-like siphophages (Figure 4F). The cryo-EM density of the distal region of gp23 was insufficient for complete atomic model building, presumably owing to intrinsic flexibility. To complement this unresolved region, an AlphaFold3 prediction of full-length gp23 was generated and used as a structural reference for subsequent comparison (Figure 4G). The distal β-rich region comprises two subdomains: a proximal β-barrel domain, which was resolved in the cryo-EM density and consists of a compact β-sheet arrangement (Figure 4F), and a distal β-scaffold domain, which could not be resolved in the cryo-EM map (Figure 4H). The corresponding region of the AlphaFold3-predicted model was subsequently used as a query for DALI searches. DALI analysis identified the FCCH domain of ubiquitin E1 enzymes (PDB 9QIM) as the top hit (DALI Z = 6.4, RMSD = 2.4 Å). However, the limited alignment length and the absence of overall structural correspondence suggest that this match reflects only local topological similarity rather than a conserved fold. Consequently, no structurally related phage or cellular protein could be identified for the distal β-rich domain.

Within the tail tip lumen, the N-terminal region of the HDIII domain of gp23 adopts a tripod-like configuration and is situated immediately below the C-terminus of TMP gp19. This region exhibits structural resemblance to the lumenal gpI assembly of lambda (Figure 4I), implying a conserved functional role. Three copies of this N-terminal region oligomerize into a trimeric support that stabilizes the TMP coiled-coil terminus within the lumen [6]. Analogous to the gpI-mediated plug system of lambda, this trimeric assembly serves to retain the TMP and to prevent the premature release of both TMP and packaged DNA from the tail tube.

The insertion protein gp21 constitutes another conserved component of the Avs-1 tail tip core and trimerizes beneath the distal tail protein (Figure S6D). Structural comparison revealed that gp21 is highly similar to lambda gpL (DALI Z = 11.1, RMSD = 2.9 Å) (Figure S6E), indicating the conservation of the insertion module among siphophages, despite low sequence identity. Protein gp21 contains an N-terminal β-sandwich domain (residues 3–159) (Figure 4J), which corresponds to the HDI domain as defined by the structural domain nomenclature for T5 and lambda tail proteins [6,30]. This domain closely resembles the HDI domain of lambda gpL, consistent with the conserved β-sandwich topology observed among siphophage tail proteins [6]. Together with the hub protein, both HDI and HDIV adopt a TTP fold, thereby extending the tail tube with pseudo-6-fold symmetry (Figure 4K). Distal to the β-sandwich core, gp21 possesses an elongated linker that ends in a short C-terminal α-helix (residues 160–171) (Figure 4J), a feature that closely mirrors the architecture of lambda gpL. However, in contrast to lambda gpL, gp21 lacks the C-terminal iron-binding domain and the characteristic metal-coordinating residues (Figure S6E).

The Avs-1 tail tip core preserves the characteristic DTP–hub–TMP framework found in lambda-like siphophages while adopting a distinct organization that produces a tripod-like architecture. Unlike the extended adsorption architecture of lambda gpJ, in which the receptor-binding domain is positioned at the distal region of the central fiber [34], Avs-1 gp23 forms a more compact structural platform within the tail tip. Similar tripod-like architectures have also been observed in other siphophages, including Chi [16], JBD30 [15], and R4C [29], although the molecular compositions and domain organizations underlying these threefold assemblies vary considerably among phages. Structural studies of JBD30 have suggested that coordinated organization of distal tail-tip components is important for maintaining an infection-competent architecture and enabling structural transitions during host engagement [15]. Given that Avs-1 gp23 lacks recognizable structural features corresponding to reported receptor-binding modules, such as Ig-like or OB-fold-containing domains, this distinct architectural organization may instead contribute to maintaining the structural integrity of the distal tail assembly during host interaction. The divergence of this region may reflect different evolutionary trajectories among siphophage lineages or distinct functional constraints associated with host recognition and tail-tip remodeling during infection [6,13,41].

### 3.5. Structure of the Trimeric Tail Spike

**Figure 5:**
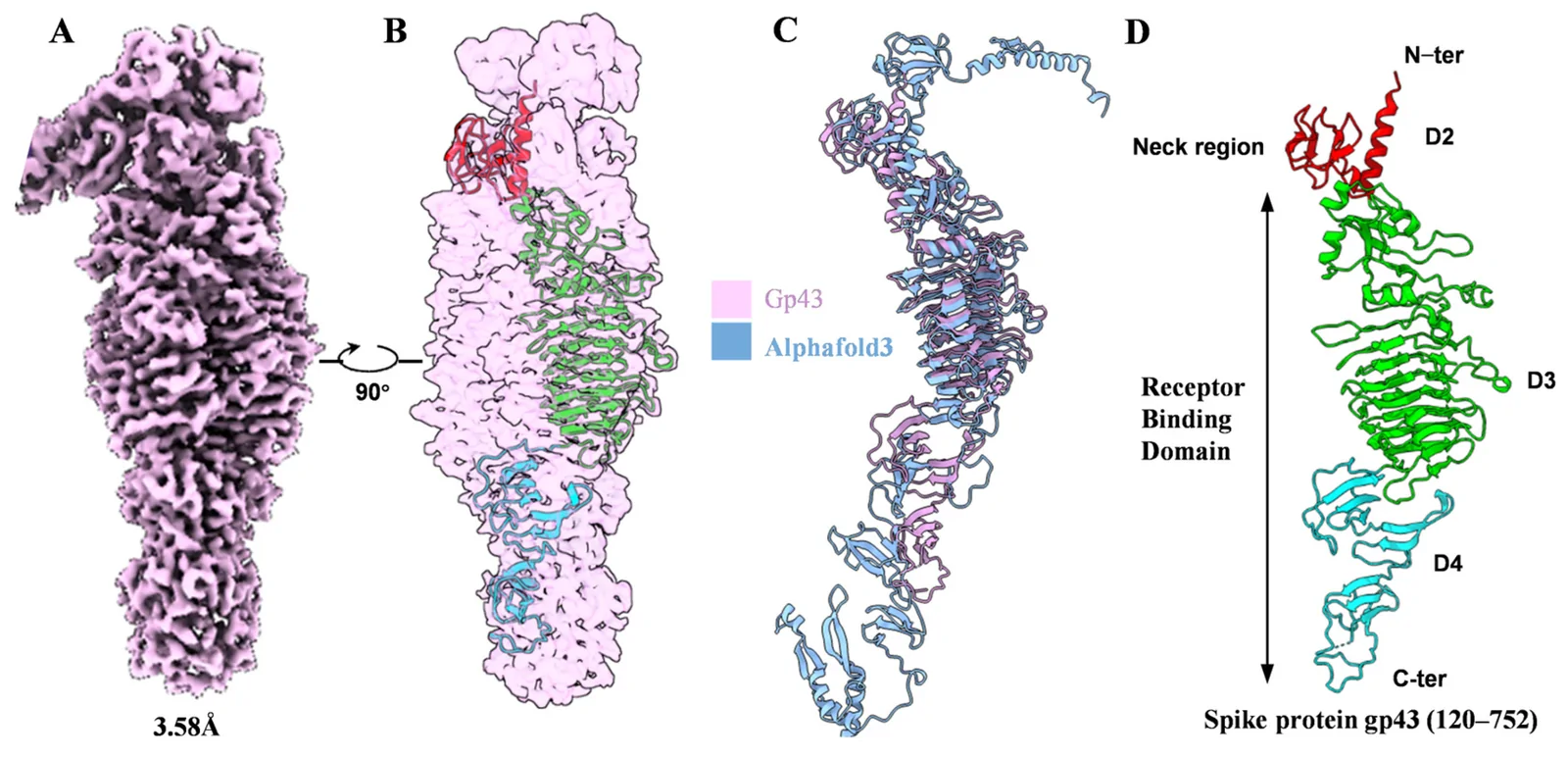
Structure of the spike protein: (**A**) Side view of the density maps of the tail spike. (**B**) Side view of the ribbon model of the spike protein gp43. (**C**) Structural superposition of our gp43 model with the AlphaFold3 prediction of full-length gp43. (**D**) Ribbon model of gp43, with distinct domains shown in different colors.

The spike protein gp43 of Avs-1 extends from the distal tail protein and oligomerizes into a trimer, constituting an integral component of the distal adsorption apparatus. This reconstruction represents the in situ architecture of the spike, offering direct insight into its arrangement within the tail tip (Figure 5A). The density map is well resolved in the central region, whereas the terminal regions exhibit low local resolution, precluding model building of the N-terminal (~residues 1–53) and the C-terminal (~residues 777–888) regions (Figure 5B). Superposition of the experimentally determined model with the AlphaFold3 prediction of full-length gp43 revealed strong agreement throughout the resolved central region. The unresolved regions correspond to elongated terminal extensions projecting from both ends of the spike, thereby illustrating the overall morphology and additional length of the full-length spike (Figure 5C). 

Based on the domain organization established for CBA120 TSPs [42], the built model encompasses the D2, D3, and a part of the D4 domains (Figure 5D). The D2 domain is predominantly composed of an α-helix that forms the neck region of the spike. The D3 domain adopts canonical β-helical folds that closely resemble those of the GVE2 spike protein [9], CBA120 TSP4 [42], P22 tailspike protein [7], and CBA120 TSP1/TSP3 [10,43]. Structural comparison using DALI revealed that the D2 and D3 domains of gp43 exhibit strong structural similarity to GVE2 (Z = 7.4, RMSD = 3.44 Å) and CBA120 TSP4 (Z = 8.9, RMSD = 3.22 Å), despite low sequence identities of 12.3% and 13.6% in the modeled portion of gp43, respectively (Figure S7). Additional structural homologs, including the P22 tailspike protein and CBA120 TSP1/TSP3, are summarized in Table S1. These results indicate that gp43 shares a conserved architectural organization with the tailspike proteins of GVE2, CBA120 TSP1, CBA120 TSP3, CBA120 TSP4, and P22. 

In contrast to the highly conserved D2 and D3 domains, the D4 domain exhibits greater structural divergence and protrudes from the distal end of the β-helical scaffold. Although sequence comparison indicates comparable similarity to GVE2 and CBA120 TSP4, structural inspection suggests that only limited local similarity exists between the D4 region and the corresponding region of the GVE2 spike protein, whereas no clear structural correspondence is evident with CBA120 TSP4. Collectively, these results suggest that diversification is primarily concentrated within the distal portion of the spike.

The in situ reconstruction of gp43 further highlights the functional specialization of the distal tail region. The spike maintains a conserved trimeric β-helical architecture while exhibiting marked structural divergence within the distal D4 region. Together with the tripod-like organization of the tail tip, this architecture highlights the distal tail region as a major site of structural diversification in Avs-1. We propose that the trimeric spike may participate in initial, reversible contacts with the bacterial surface, followed by more stable attachment mediated through coordinated interactions among multiple tail-tip components, as suggested by structural studies of other siphophages [10,15,16,42,43]. Although the precise receptor and functional mechanisms remain to be determined, the distinct organization of the tail tip and spike provides a structural framework for investigating their potential roles in phage–host interactions. Overall, these findings demonstrate that Avs-1 combines a conserved virion framework with diversified distal modules, providing a structural basis for further investigating phage–host interactions in aquatic environments.

## Data Availability

The complete genome sequences of AV1212 have been submitted to GenBank under accession number CP196963. The complete genome sequence of phage Avs-1 has been uploaded to GenBank under accession number PX024133. The complete genomes of AV1212 and Avs-1 raw sequence reads have been submitted to the Sequence Read Archive (SRA) under accession numbers SRR37013324 and SRR37018936. Cryo-EM maps and the structures have been deposited at the Electron Microscopy Data Bank (EMD-67163, EMD-81923, EMD-81927, EMD-67166, and EMD-67168) and the Protein Data Bank (PDB ID: 43JK, 43JA, 43JB, 43JC, 43JD, 43JE, 43JF, 43JG, 43JH, 43JI, and 43JJ) and are publicly available as of the date of publication.

## Supplementary Materials

The following supporting information can be downloaded at: https://www.mdpi.com/article/10.3390/v18091011/s1, Figure S1: Cryo-EM images and Fourier shell correlation curves of siphophage Avs-1; Figure S2: Local resolution maps of Avs-1 from the head to the tip; Figure S3: Quality of the cryo-EM density maps and the atomic models of the 12 proteins of Avs-1; Figure S4: Structures of the MCP and CP of Avs-1; Figure S5: Structural comparison of the portal-neck complex and the tail tube in different siphophages; Figure S6: Structures of the tip core; Figure S7: Structural comparisons of the spike protein of Avs-1 with spike proteins of GVE2, P22, and CBA120 TSP4/TSP3/TSP1; Table S1: Structural differences of spike proteins among GVE2, CBA120 and P22 using Avs-1 as the reference; Table S2: Refinement and model statistics.

## References

[1] Bartie K.L., Desbois A.P. (2024). *Aeromonas dhakensis*: A Zoonotic Bacterium of Increasing Importance in Aquaculture. Pathogens.

[2] Park S.Y., Han J.E., Kwon H., Park S.C., Kim J.H. (2020). Recent Insights into *Aeromonas salmonicida* and Its Bacteriophages in Aquaculture: A Comprehensive Review. J. Microbiol. Biotechnol..

[3] Igbinosa I.H., Beshiru A., Odjadjare E.E., Ateba C.N., Igbinosa E.O. (2017). Pathogenic potentials of *Aeromonas* species isolated from aquaculture and abattoir environments. Microb. Pathog..

[4] Arisaka F., Yap M.L., Kanamaru S., Rossmann M.G. (2016). Molecular assembly and structure of the bacteriophage T4 tail. Biophys. Rev..

[5] Flayhan A., Vellieux F.M., Lurz R., Maury O., Contreras-Martel C., Girard E., Boulanger P., Breyton C. (2014). Crystal structure of pb9, the distal tail protein of bacteriophage T5: A conserved structural motif among all siphophages. J. Virol..

[6] Xiao H., Tan L., Tan Z., Zhang Y., Chen W., Li X., Song J., Cheng L., Liu H. (2023). Structure of the siphophage neck-Tail complex suggests that conserved tail tip proteins facilitate receptor binding and tail assembly. PLoS Biol..

[7] Iglesias S.M., Lokareddy R.K., Yang R., Li F., Yeggoni D.P., David Hou C.F., Leroux M.N., Cortines J.R., Leavitt J.C., Bird M. (2023). Molecular Architecture of Salmonella Typhimurium Virus P22 Genome Ejection Machinery. J. Mol. Biol..

[8] Ge X., Wang J. (2024). Structural mechanism of bacteriophage lambda tail’s interaction with the bacterial receptor. Nat. Commun..

[9] Zhang L., Yan Y., Gan Q., She Z., Zhu K., Wang J., Gao Z., Dong Y., Gong Y. (2020). Structural and functional characterization of the deep-sea thermophilic bacteriophage GVE2 tailspike protein. Int. J. Biol. Macromol..

[10] Chen C., Bales P., Greenfield J., Heselpoth R.D., Nelson D.C., Herzberg O. (2014). Crystal structure of ORF210 from *E. coli* O157:H1 phage CBA120 (TSP1), a putative tailspike protein. PLoS ONE.

[11] Li F., Hou C.D., Yang R., Whitehead R., Teschke C.M., Cingolani G. (2022). High-resolution cryo-EM structure of the Shigella virus Sf6 genome delivery tail machine. Sci. Adv..

[12] Chen Y., Xiao H., Zhou J., Peng Z., Peng Y., Song J., Zheng J., Liu H. (2025). The In Situ Structure of T-Series T1 Reveals a Conserved Lambda-Like Tail Tip. Viruses.

[13] Linares R., Arnaud C.A., Effantin G., Darnault C., Epalle N.H., Boeri Erba E., Schoehn G., Breyton C. (2023). Structural basis of bacteriophage T5 infection trigger and *E. coli* cell wall perforation. Sci. Adv..

[14] Peng Y., Pang H., Zheng J., Zhou J., Chen W., Yang F., Xiao H., Liu H. (2026). Structure of a Brochothrix thermosphacta bacteriophage reveals cell wall adsorption mechanism in Gram-positive infecting siphophages. Nat. Commun..

[15] Valentová L., Füzik T., Nováček J., Hlavenková Z., Pospíšil J., Plevka P. (2024). Structure and replication of Pseudomonas aeruginosa phage JBD30. EMBO J..

[16] Sonani R.R., Esteves N.C., Scharf B.E., Egelman E.H. (2024). Cryo-EM structure of flagellotropic bacteriophage Chi. Structure.

[17] Doss J., Culbertson K., Hahn D., Camacho J., Barekzi N. (2017). A Review of Phage Therapy against Bacterial Pathogens of Aquatic and Terrestrial Organisms. Viruses.

[18] Tekedar H.C., Kumru S., Blom J., Perkins A.D., Griffin M.J., Abdelhamed H., Karsi A., Lawrence M.L. (2019). Comparative genomics of *Aeromonas veronii*: Identification of a pathotype impacting aquaculture globally. PLoS ONE.

[19] Xu X., Fu H., Wan G., Huang J., Zhou Z., Rao Y., Liu L., Wen C. (2022). Prevalence and genetic diversity of *Aeromonas veronii* isolated from aquaculture systems in the Poyang Lake area, China. Front. Microbiol..

[20] Gallage T.P., Paisantham P., Surachetpong W., Mongkolsuk S., Sirikanchana K. (2025). Prospects of Phage DJ6712 and FW6709 in Biocontrol of *Aeromonas veronii* in Fish Aquaculture. Microorganisms.

[21] Li C., Liu J., Long X., Qian L., Xie J., Luo C., Yang X., He Y., Zhang Y., Cai H. (2026). Isolation and characterization of Avs-1, a bacteriophage effective against the aquaculture pathogen *Aeromonas veronii*. Appl. Environ. Microbiol..

[22] Punjani A., Rubinstein J.L., Fleet D.J., Brubaker M.A. (2017). cryoSPARC: Algorithms for rapid unsupervised cryo-EM structure determination. Nat. Methods.

[23] Jamali K., Käll L., Zhang R., Brown A., Kimanius D., Scheres S.H.W. (2024). Automated model building and protein identification in cryo-EM maps. Nature.

[24] Emsley P., Lohkamp B., Scott W.G., Cowtan K. (2010). Features and development of Coot. Acta Crystallogr. D Biol. Crystallogr..

[25] Adams P.D., Afonine P.V., Bunkóczi G., Chen V.B., Davis I.W., Echols N., Headd J.J., Hung L.W., Kapral G.J., Grosse-Kunstleve R.W. (2010). PHENIX: A comprehensive Python-based system for macromolecular structure solution. Acta Crystallogr. D Biol. Crystallogr..

[26] Kucukelbir A., Sigworth F.J., Tagare H.D. (2014). Quantifying the local resolution of cryo-EM density maps. Nat. Methods.

[27] Wikoff W.R., Liljas L., Duda R.L., Tsuruta H., Hendrix R.W., Johnson J.E. (2000). Topologically linked protein rings in the bacteriophage HK97 capsid. Science.

[28] Li F., Hou C.D., Lokareddy R.K., Yang R., Forti F., Briani F., Cingolani G. (2023). High-resolution cryo-EM structure of the Pseudomonas bacteriophage E217. Nat. Commun..

[29] Huang Y., Sun H., Wei S., Cai L., Liu L., Jiang Y., Xin J., Chen Z., Que Y., Kong Z. (2023). Structure and proposed DNA delivery mechanism of a marine roseophage. Nat. Commun..

[30] Peng Y., Tang H., Xiao H., Chen W., Song J., Zheng J., Liu H. (2024). Structures of Mature and Urea-Treated Empty Bacteriophage T5: Insights into Siphophage Infection and DNA Ejection. Int. J. Mol. Sci..

[31] Zhou J., Wang L., Xiao H., Chen W., Liu Z., Song J., Zheng J., Liu H. (2025). In situ structures of the contractile nanomachine myophage Mu in both its extended and contracted states. J. Virol..

[32] Ayala R., Moiseenko A.V., Chen T.H., Kulikov E.E., Golomidova A.K., Orekhov P.S., Street M.A., Sokolova O.S., Letarov A.V., Wolf M. (2023). Nearly complete structure of bacteriophage DT57C reveals architecture of head-to-tail interface and lateral tail fibers. Nat. Commun..

[33] Bárdy P., Füzik T., Hrebík D., Pantůček R., Thomas Beatty J., Plevka P. (2020). Structure and mechanism of DNA delivery of a gene transfer agent. Nat. Commun..

[34] Wang C., Duan J., Gu Z., Ge X., Zeng J., Wang J. (2024). Architecture of the bacteriophage lambda tail. Structure.

[35] Chagot B., Auzat I., Gallopin M., Petitpas I., Gilquin B., Tavares P., Zinn-Justin S. (2012). Solution structure of gp17 from the Siphoviridae bacteriophage SPP1: Insights into its role in virion assembly. Proteins.

[36] Yang F., Jiang Y.L., Zhang J.T., Zhu J., Du K., Yu R.C., Wei Z.L., Kong W.W., Cui N., Li W.F. (2023). Fine structure and assembly pattern of a minimal myophage Pam3. Proc. Natl. Acad. Sci. USA.

[37] Yang F., Wang L., Zhou J., Xiao H., Liu H. (2023). In Situ Structures of the Ultra-Long Extended and Contracted Tail of Myoviridae Phage P1. Viruses.

[38] Ge P., Scholl D., Prokhorov N.S., Avaylon J., Shneider M.M., Browning C., Buth S.A., Plattner M., Chakraborty U., Ding K. (2020). Action of a minimal contractile bactericidal nanomachine. Nature.

[39] Jiang F., Li N., Wang X., Cheng J., Huang Y., Yang Y., Yang J., Cai B., Wang Y.P., Jin Q. (2019). Cryo-EM Structure and Assembly of an Extracellular Contractile Injection System. Cell.

[40] Orlov I., Roche S., Brasilès S., Lukoyanova N., Vaney M.C., Tavares P., Orlova E.V. (2022). CryoEM structure and assembly mechanism of a bacterial virus genome gatekeeper. Nat. Commun..

[41] Veesler D., Cambillau C. (2011). A common evolutionary origin for tailed-bacteriophage functional modules and bacterial machineries. Microbiol. Mol. Biol. Rev..

[42] Plattner M., Shneider M.M., Arbatsky N.P., Shashkov A.S., Chizhov A.O., Nazarov S., Prokhorov N.S., Taylor N.M.I., Buth S.A., Gambino M. (2019). Structure and Function of the Branched Receptor-Binding Complex of Bacteriophage CBA120. J. Mol. Biol..

[43] Greenfield J., Shang X., Luo H., Zhou Y., Heselpoth R.D., Nelson D.C., Herzberg O. (2019). Structure and tailspike glycosidase machinery of ORF212 from E. coli O157:H7 phage CBA120 (TSP3). Sci. Rep..

