# Supplementary material for: Cryo-EM Structure of Siphophage Avs-1 Reveals a Conserved Virion Architecture with a Specialized Tripod-like Tail Tip for Host Recognition
Source: Viruses. 2026 Sep 14;18(9):1011. doi: 10.3390/v18091011 (PMC13612043; doi:10.3390/v18091011)
Supplement: Supplementary file 1 [file viruses-18-01011-s001.zip › viruses-4480055-supplementary.pdf]

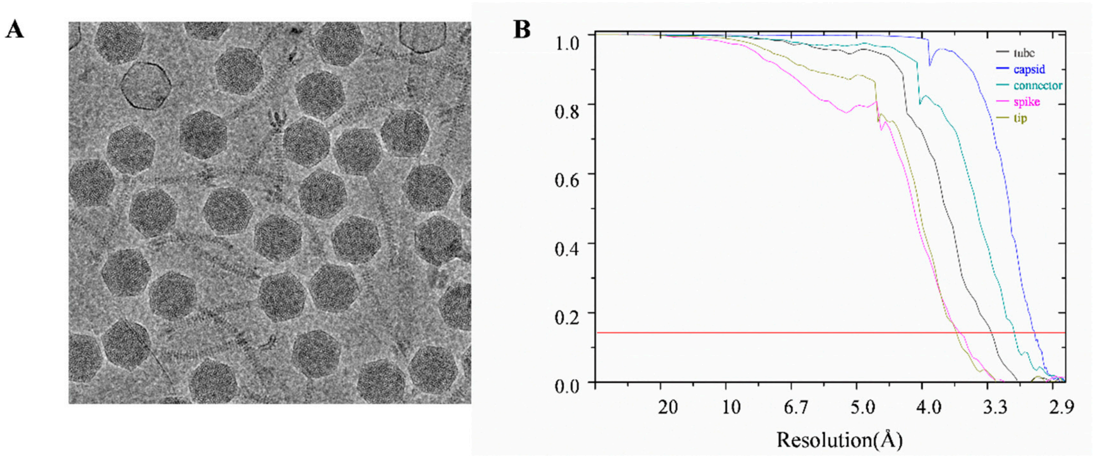

Figure S1. Cryo-EM images and Fourier shell correlation curves of siphophage Avs-1. **(A)** Representative cryo-EM image of Avs-1. **(B)** Estimated structural resolutions of the icosahedral head (blue), connector (dark cyan), tail tube (black), tail tip (dark yellow) and tail spike (magenta) of Avs-1.

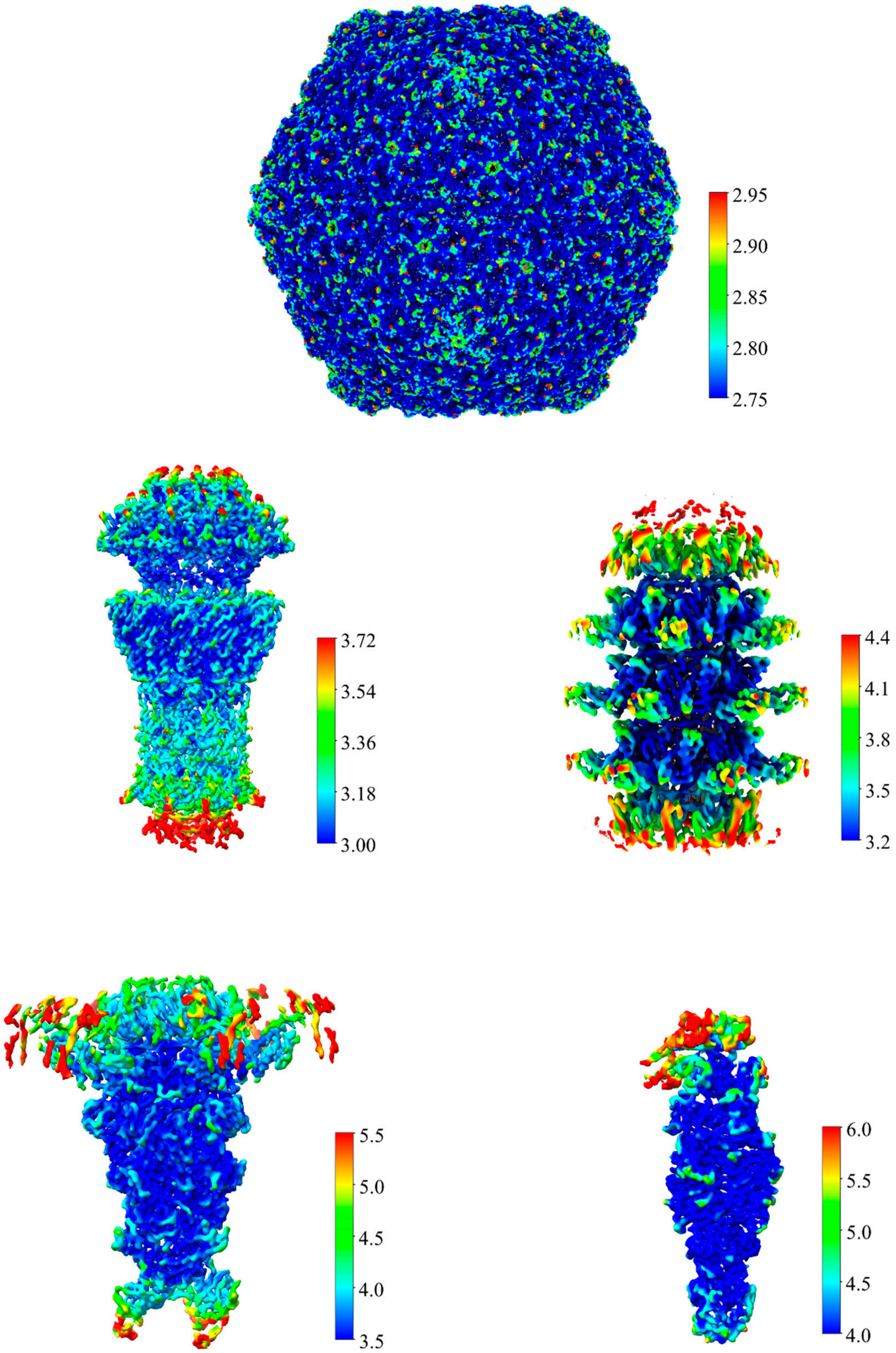

Figure S2. Local resolution maps of the Avs-1 from the head to the tip.

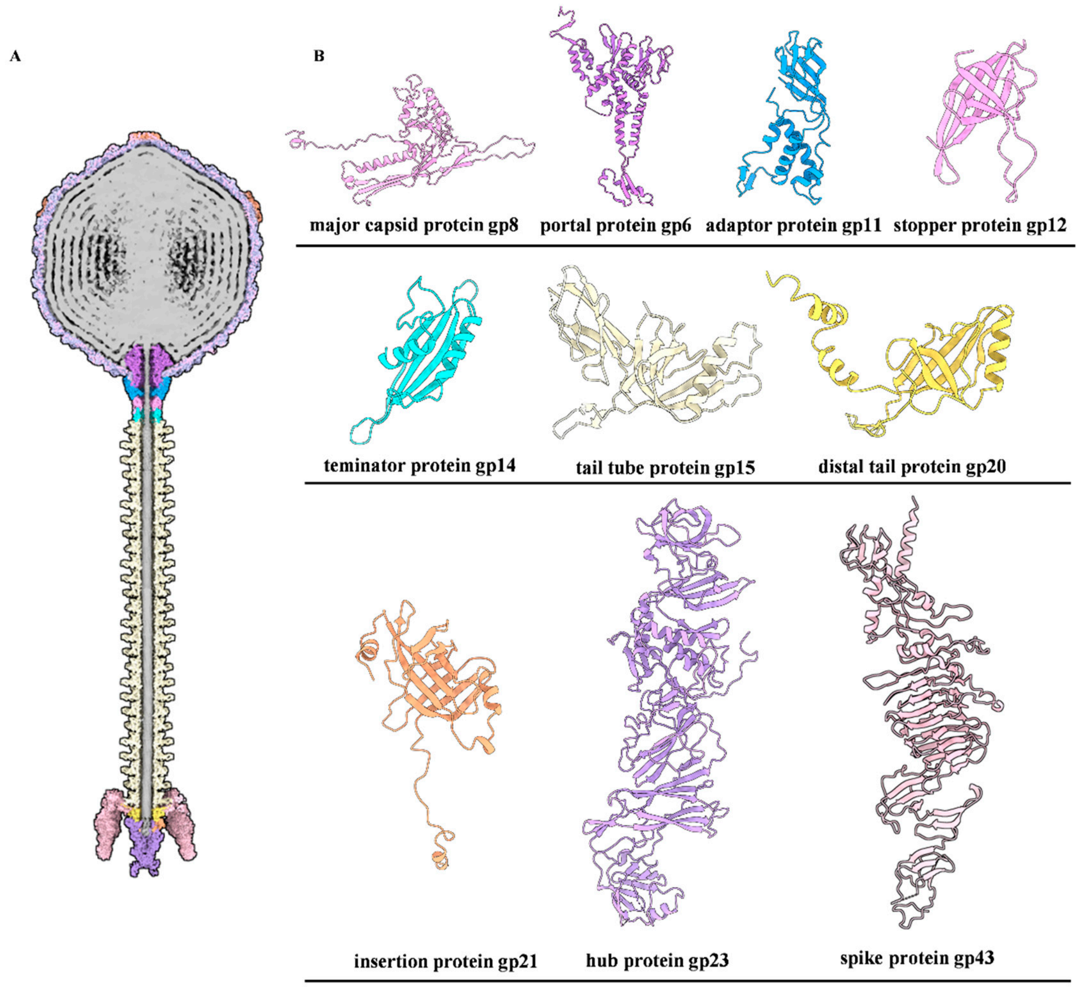

Figure S3. Quality of the cryo-EM density maps and the atomic models of the 12 proteins of Avs-1. **(A)** Cut-open view of the asymmetric structure of Avs-1. All color codes are identical to those used in Fig. 1A. **(B)** Ribbon models of 13 proteins from the head to the tail tip.

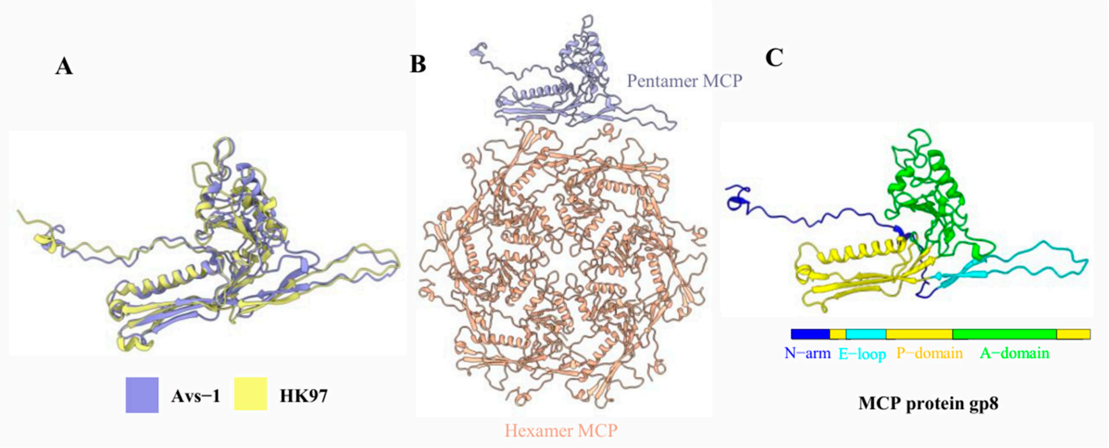

Figure S4. Structures of the MCP and CP of Avs-1. **(A)** Structural comparison of the ribbon models between the Avs-1 MCP gp8 and the HK97 MCP gp5 (PDB ID: 1OHG). **(B)** Ribbon models of an asymmetric unit of capsid, and gp8 are colored in light purple and light orange, respectively. **(C)** Ribbon representation of the MCP gp8 structure, showing its four defined domains: the N-terminal arm (N-arm, residues 132–176), peripheral domain (P-domain, residues 212–268 and 404–437), extended loop (E-loop, residues 177–211), and axis domain (A-domain, residues 269–403 and 438–453).

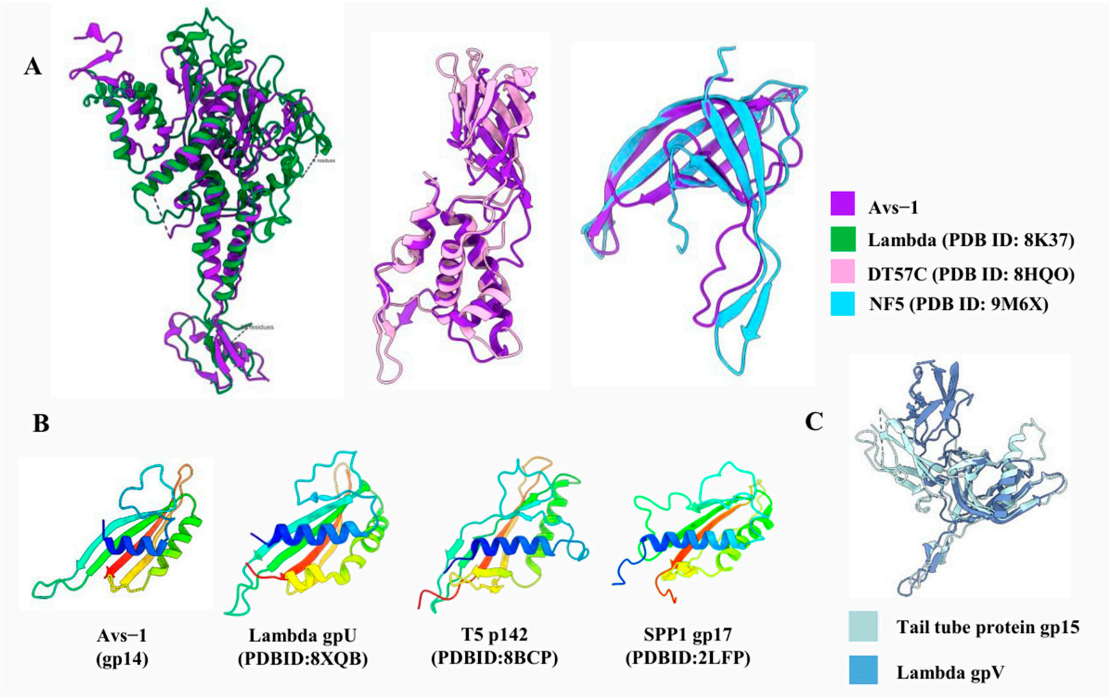

Figure S5. Structural comparison of the portal-neck complex and the tail tube in different siphophages. **(A)** Structural comparisons of the portal and neck complex between siphophages Avs-1, Lambda, DT57C and NF5. **(B)** Structural comparisons of terminator protein of Avs-1, as well as terminator proteins of other siphophages. Gp14 in Avs-1, gpU in Lambda, p142 in T5 and gp17 in SPP1 are shown in rainbow colors. **(C)** Structural comparison of tail tube protein between Avs-1 and lambda (PDB ID: 8iyk).

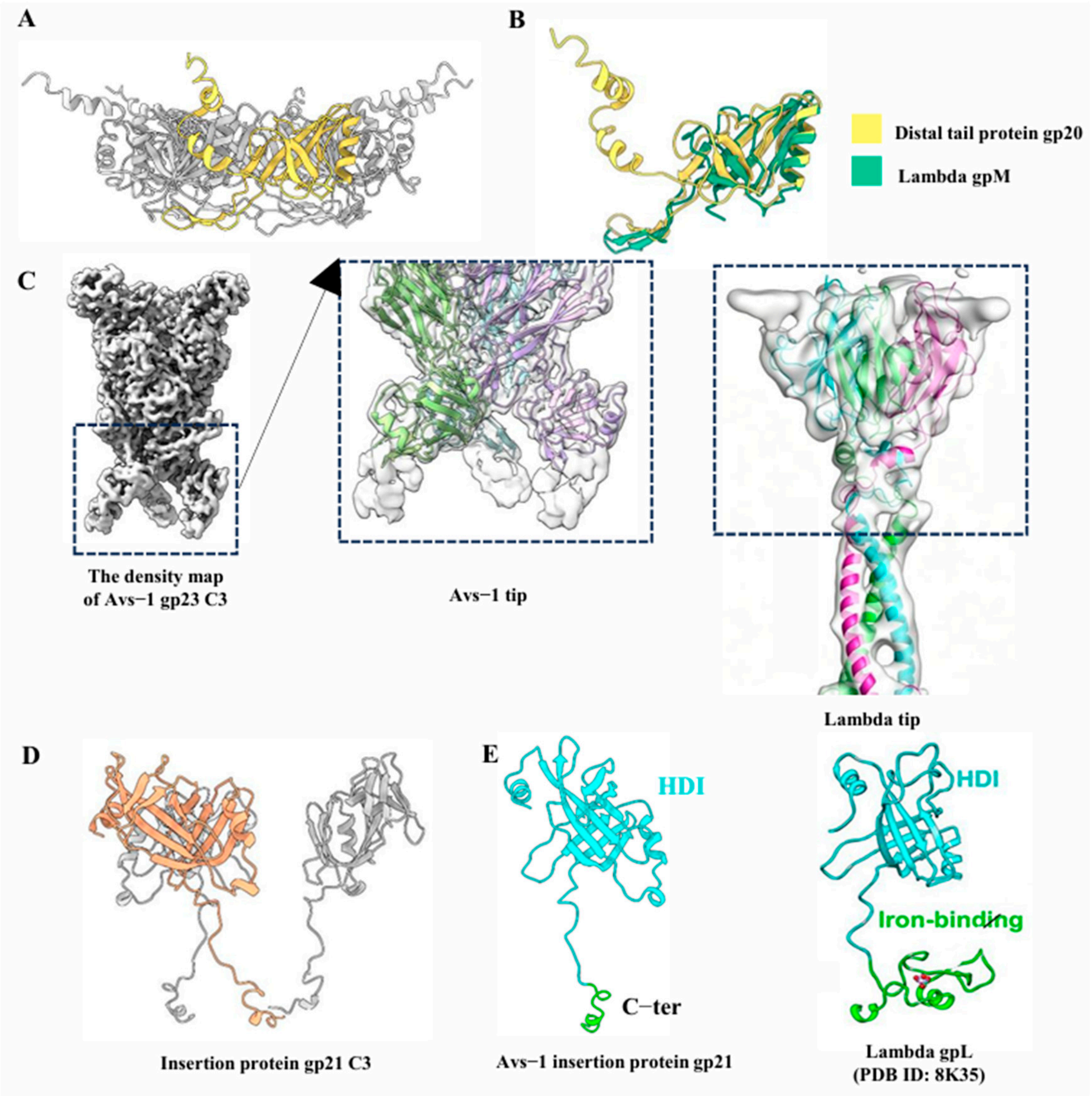

Figure S6. Structures of the tip core. **(A)** Side view of a hexamer of distal tail protein gp20. **(B)** Structural comparison of distal tail protein between Avs-1 and lambda. **(C)** The density map comparison of the Avs-1 and lambda tail tips. Unlike lambda gpJ, the distal region of Avs-1 gp23 exhibits a distinct organization without the α-helical shaft, central straight fiber, or receptor-binding domain. **(D)** Side view of a trimer of gp21. **(E)** Structural comparison of insertion proteins between Avs-1 and lambda, colored according to their domains.

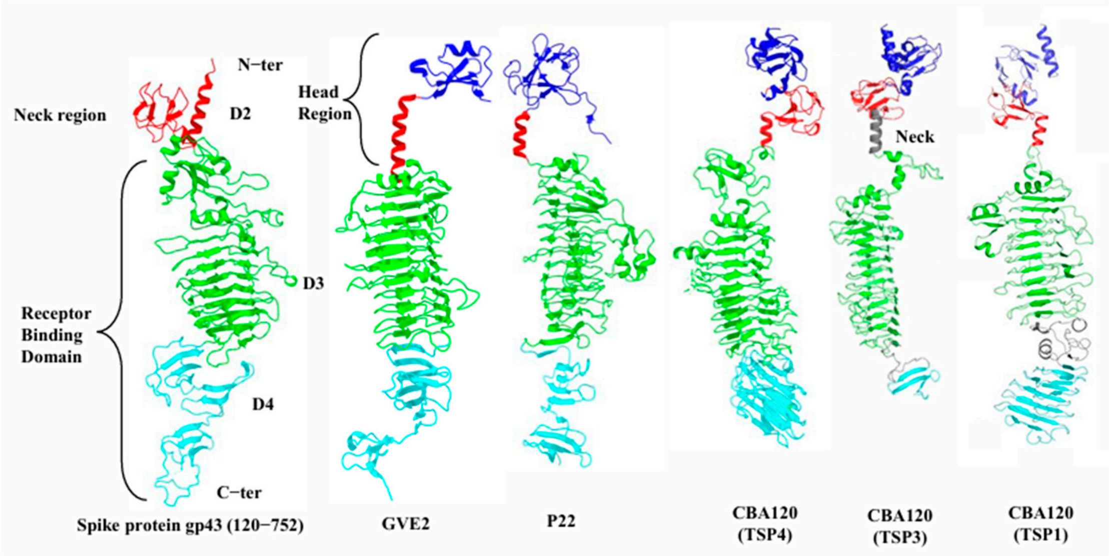

Figure S7. Structural comparisons of spike protein of Avs-1, as well as spike proteins of GVE2, P22, and CBA120 TSP4/TSP3/TSP1.

Table S1. The structural differences of spike proteins among GVE2, CBA120 and P22. Using Avs-1 as the reference.

Protein	Z, Avs−1 reference	RMSD, Avs−1 reference
Avs−1 gp43	–	–
GVE2 TSP	7.4	3.44 Å
CBA120 TSP4	8.9	3.22 Å
P22 gp9	7.4	3.78 Å
CBA120 TSP1/TSP3	7.8−7.9	~ 3.2 Å

Table S2. Refinement and model statistics

Data collection											
Electron microscopy	TOTEN 300s, Gantan K3 camera										
Pixel size((Å))	1.248										
Defocus range	0										
Total movie-mode micrographs	Avs-1										
	3,068										
**Local reconstruction**											
	Capsid	Portal-neck				Tail tube	Tail tip core				Tail tip spike
Total particles	44,943	44,943				44,930	22,840				22,840
Resolution(Å)	2.98	3.12				3.3	3.62				3.58
B-factors	139.5	90.1				116.1	87.3				111.6
EMDB ID	EMD-67163	EMD-81923				EMD-81927	EMD-67166				EMD-67168
**Atomic models refinement/statistics (phenix.real_space_refine)**											
	Capsid	Portal-neck				Tail tube	Tail tip core				Tail tip spike
Protein	gp8	gp6	gp11	gp12	gp14	gp15	gp19	gp20	gp21	gp23	gp43
PDB ID	43JK	43JA	43JB	43JC	43JD	43JE	43JF	43JG	43JH	43JI	43JJ
Model Resolution in Refinement (Å)	2.98	3.12				3.3	3.62				3.58
Total Residues	2248	361	168	103	116	217	45	159	169	753	1881
CC (model to map fit)	0.8781	0.6838	0.7083	0.6884	0.7038	0.7262	0.7863	0.7544	0.7109	0.8026	0.7443
Ramachandran most favorable (%)	96.15	95.82	98.8	99.01	96.49	93.36	97.67	95.54	94.01	91.54	93.16
Ramachandran additionally allowed (%)	3.81	4.18	1.2	0.99	3.51	6.64	0	3.18	5.99	8.19	6.79
Ramachandran disallowed (%)	0.04	0	0	0	0	0	2.33	1.27	0	0.27	0.05
